# Transient Decline in Hippocampal Theta Activity during the Acquisition Process of the Negative Patterning Task

**DOI:** 10.1371/journal.pone.0070756

**Published:** 2013-07-31

**Authors:** Yuya Sakimoto, Kana Okada, Kozue Takeda, Shogo Sakata

**Affiliations:** 1 Graduate School of Medicine, Yamaguchi University, Ube-shi, Yamaguchi-ken, Japan; 2 Graduate School of Integrated Arts and Sciences, Hiroshima University, Higashihiroshima-shi, Hiroshima-ken, Japan; Pennsylvania State University, United States of America

## Abstract

Hippocampal function is important in the acquisition of negative patterning but not of simple discrimination. This study examined rat hippocampal theta activity during the acquisition stages (early, middle, and late) of the negative patterning task (A+, B+, AB-). The results showed that hippocampal theta activity began to decline transiently (for 500 ms after non-reinforced stimulus presentation) during the late stage of learning in the negative patterning task. In addition, this transient decline in hippocampal theta activity in the late stage was lower in the negative patterning task than in the simple discrimination task. This transient decline during the late stage of task acquisition may be related to a learning process distinctive of the negative patterning task but not the simple discrimination task. We propose that the transient decline of hippocampal theta activity reflects inhibitory learning and/or response inhibition after the presentation of a compound stimulus specific to the negative patterning task.

## Introduction

The hippocampus plays an important role in responding to conflicting stimuli between incompatible goals or response tendencies. Specifically, the hippocampus plays a role in increasing the weight of negative information, thereby inhibiting response to a conflicting stimulus. Chan et al. [1] and Davidson and Jarrard [2] proposed that, in association learning, response inhibition to a conflicting stimulus occurred when a stimulus had simple inhibitory associations between events embedded in concurrent simple excitatory associations, and that the hippocampus played an important role in the formation of simple inhibitory associations. They described a task that required the formation of a simple inhibitory association during inhibitory learning and proposed that negative patterning tasks typically involve inhibitory learning, whereas simple discrimination tasks typically involve non-inhibitory learning. In a negative patterning task (A+, B+, AB-), animals’ responses for 2 single stimuli (A+/B+) are reinforced and that for the compound stimulus (AB-) is not reinforced. In a simple discrimination task (A+, B-), animals’ responses for 1 stimulus (A+) are reinforced and that for the other stimulus (B-) are not. Chan et al. [1] stated that the hippocampal function was important for the no-go response to the compound stimulus in a negative patterning task, but not for the no-go response to the non-reinforced stimulus in a simple discrimination task. In support of this, several hippocampal lesion studies have shown that hippocampal function is important for the acquisition of a negative patterning task [3–5]. For example, Sutherland and Rudy [5] showed that kainic- or colchicine-induced lesions of the hippocampus impaired acquisition of a negative patterning task in rats, resulting in the inability of these rats to learn the proper response for a compound stimulus. In contrast, rats with hippocampal lesions were able to learn a simple discrimination task. Results from other studies supported the finding that hippocampal function is important for the acquisition of a negative patterning task, but not of a simple discrimination task [3,4].

The hippocampal theta activity was recorded from the hippocampal CA1. Previous studies have examined the relationship between hippocampal theta activity and acquisition of learning tasks [6,7]. Grastyán et al. [6] showed increased hippocampal theta activity during acquisition of the association between stimulus and orientative (conditioned) response in cats. In addition, several recent studies have investigated the relationship between hippocampal theta activity and the acquisition of hippocampal-dependent learning tasks such as place learning [8–11], contextual fear conditioning [12] and configural task [13,14] in rat.

Recently Sakimoto et al [14] examined the hippocampal theta activity during retention of negative patterning. However, to our knowledge, no previous study focused on the change in hippocampal theta activity with acquisition of negative patterning task. Therefore, in this study, we observed the hippocampal theta activity during acquisition of a negative patterning task in order to explore the relationship between hippocampal theta activity and the development of inhibitory learning.

## Materials and Methods

### Ethics statement

This study was carried out in strict accordance with the recommendations in the Guide for the Care and Use of Laboratory Animals of the National Institutes of Health. The protocol was approved by the Committee on the Ethics of Animal Experiments of the University of Hiroshima (Permit Number: C12-4). All surgery was performed under sodium thiamylal pentobarbital anesthesia, and all efforts were made to minimize suffering.

### Animals

Twelve naïve, 3-month-old male Wistar albino rats were used in this study. All rats were individually housed and maintained on a 12: 12-h light-dark cycle (lights on at 8: 00 a.m.). Throughout the experiment, water was available continuously and rats were maintained at 85% of their *ad libitum* body weights. All procedures for animal treatment and surgery were approved by the Hiroshima University guidelines for animal experiments.

### Apparatus

Behavioral training and electroencephalogram (EEG) recording sessions were conducted in a standard operant chamber (ENV-007 CT; MED Associates, Inc., USA). The chamber was housed in a soundproof, electrically shielded room. For delivery of 45-mg food pellets (Bio Serv PRODUCT #F0165), a cup was located in the center of the front wall at floor level and a light bulb (ENV-215; MED Associates, Inc.) was mounted over the cup to provide constant illumination. A lever was positioned on the left side of the front wall. A white super luminosity LED light (41 lux) was mounted on the ceiling to present light stimulus. A tone (2000 Hz, 75 dB) was provided via a speaker placed on the interior shell. All events were controlled, and behavioral data recorded on a personal computer (EPSON MT7500).

### Procedure

A 30-min habituation session was conducted in the operant box, and then the rats were trained to press the lever to receive food pellets. After acquisition of the lever press response, rats were given 2 days of continuous reinforcement (CRF; 100 reinforcements/day), followed by 2 days of variable interval (VI) reinforcement with 20-s schedules (40 reinforcements/day). After VI 20-s training, electrodes for EEG recording were implanted in each rat. After a recovery period of at least 1 week, rats were divided into 2 groups based on performance during the VI 20-s training. One group (*n* = 6) was given training in the negative patterning task, and the another group (*n* = 6) was trained in the simple discrimination task. EEG was recorded until completion of learning of the task.

### Negative patterning task

**Figure 1:**
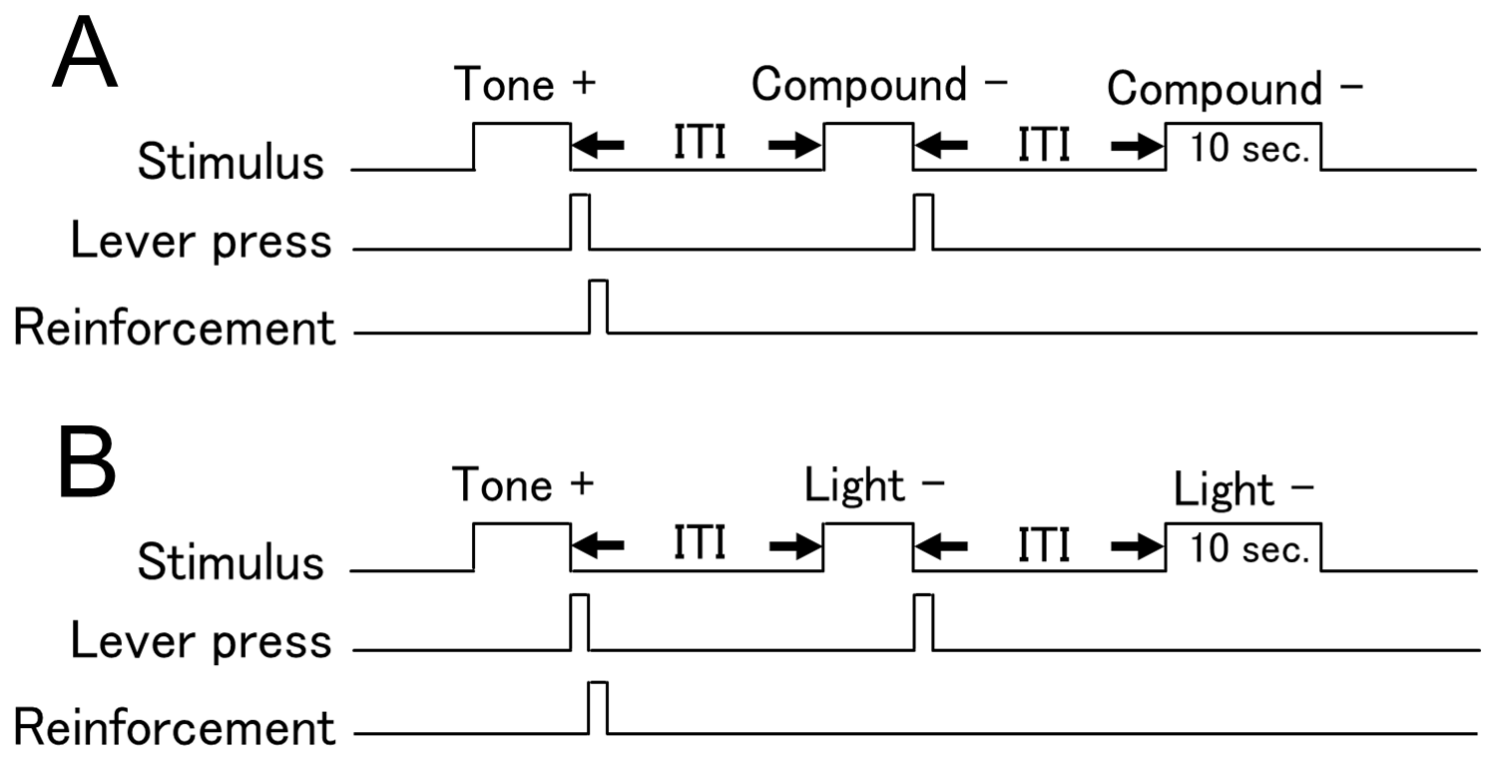
Negative patterning and simple discrimination paradigms. In the negative patterning task, lever presses were reinforced following either of the stimulus elements (Tone +, Light +), but not following the compound stimulus (Compound -; panel A). In the simple discrimination task, for 1 group, lever responses were rewarded when the tone stimulus was presented (Tone +), but not when the light stimulus was presented (Light -; panel B). For the other group, the relationship between cue modality and availability of reinforcement was reversed (Light +, Tone -).

In the negative patterning discrimination task, rats were trained to discriminate simple stimuli (Tone or Light) from a compound stimulus (Tone and Light). Each session consisted of 120 trials, made up of 60 reinforcement trials (RFTs) and 60 non-reinforcement trials (Non-RFTs). In RFTs, Tone and Light were presented separately, and the rat’s lever responses were rewarded (T+, L+). In non-RFTs, Tone and Light stimulus were presented simultaneously, and the rat’s lever responses were not rewarded (TL−). All stimuli remained on until either 10 s had elapsed or the rat pressed the lever. Each trial was separated by variable intertrial intervals (ITI, 20–40 s; Figure 1A). The sequence of stimuli was randomly determined, with the constraints that no more than 4 trials of the same type occurred in succession. Response rates for RFT (Response rate for RFT = the number of the lever press for RFTs in a session/the number of total RFTs in a session) and non-RFT (Response rate for non-RFT = the number of the lever press for non-RFTs in a session/the number of total non-RFTs in a session) were calculated. Learning criteria were achieved when the discriminative rate (= response rate for RFT – response rate for non-RFT) was at least 50%. If the criteria were achieved for 2 consecutive days, learning was considered complete. In this experiment, acquisition of the tasks was divided into 3 stages (early stage: first session in discrimination training; middle stage: first session when discriminative rate was ≥ 25%; late stage: first session when discriminative rate was ≥ 50%).

### Simple discrimination task

In the simple discrimination task, rats were trained to discriminate between 2 single stimuli (Tone and Light; Figure 1B). After surgery recovery period, rats in this group were randomly assigned to 2 further groups of simple discrimination tasks. For 1 group (T+, L-), lever responses were rewarded when the tone stimulus was presented (T+), but not when the light stimulus was presented (L-). For the other group, the relationship between cue modality and availability of reinforcement was reversed (L+, T-). The rest of the protocol was the same as described for the negative patterning task.

### Electrode implantation

After they were deeply anesthetized with thiamylal sodium (50 mg/kg i.p.), the rats were placed in a stereotaxic apparatus (Narishige, Japan). EEG was recorded using the bipolar method, with the recording electrodes implanted stereotaxically in the hippocampal region, 2.4 mm below the skull surface, 3.5 mm posterior to the bregma, and ±2.0 mm lateral to the midline. The reference electrodes were attached to the skull 6.0 mm anterior to the bregma and ±2.0 mm lateral to the midline. Polyurethane-insulated stainless steel wire electrodes (200-µm diameter; Unique Medical Co., LTD., Japan) were used as the recording electrodes, and polyurethane-insulated silver-ball electrodes (1-mm diameter; Unique Medical Co., LTD., Japan) were used as the reference electrodes. The electrodes were terminated by pin-type connectors, which were connected to sockets attached to the skull over the hippocampus using anchor screws and dental cement.

### Electroencephalogram recording and analysis

EEG data from early, middle, and late learning stages were analyzed. The EEG waveforms were amplified (System 360; NEC Sanei) and digitized at a sampling rate of 1000 Hz by AD converter. The time constant was 3 s. Hippocampal theta activity was recorded using the mean theta power between 6 Hz and 12 Hz. The period of analysis lasted from 750 ms pre-stimulus to 4000 ms post-stimulus, and hippocampal theta power was computed with wavelet analysis using 2-ms bins. EEG analysis was used with the wavelet tool box (Morlet) provided within the MATLAB (MatLab 2007, The Math Works Inc., USA) signal processing toolbox to determine the power of theta oscillatory activity. The analysis period of -750 to +4000 ms was divided into 19 250-ms epochs. The period between -750 ms and -500 ms was used as the baseline (this period contained no stimuli), and the relative theta activity was calculated for each period as follows: relative theta activity of each period = theta activity of each period/theta activity during baseline. The analysis of hippocampal EEG included counting the number of correct responses for both RFTs and non-RFTs and the number of incorrect responses for non-RFTs. A go response for the reinforced stimulus was defined as the correct response for RFTs, a no-go response for the non-reinforced stimulus was defined as the correct response for non-RFTs, and a go response for the non-reinforced stimulus was defined as the incorrect response for non-RFTs in both tasks. Trials with artifacts were eliminated from the wavelet analysis.

### Statistical analysis

Behavioral data were calculated to examine the difference of response rate between RFT and non-RFT. The difference of response rate was assessed using analysis of variance (ANOVA) with learning stage (early, middle, and late) as a within-subject factor and group (negative patterning task and simple discrimination task groups) as a between-subject factor. The change of hippocampal theta activity with time course (from -750 ms to 4000 ms was divided into 19 epochs, with each 250 ms) of the negative patterning task was compared between the 3 learning stages (early, middle, and late) as within-subject factors on each trial type (RFT or non-RFT). After this analysis, we compared the hippocampal theta activity for each learning stage (early, middle, and late) of the negative patterning and simple discrimination tasks. Multiple comparison were conducted with the *Bonferroni* method (α = 0.05).

### Histology

**Figure 2:**
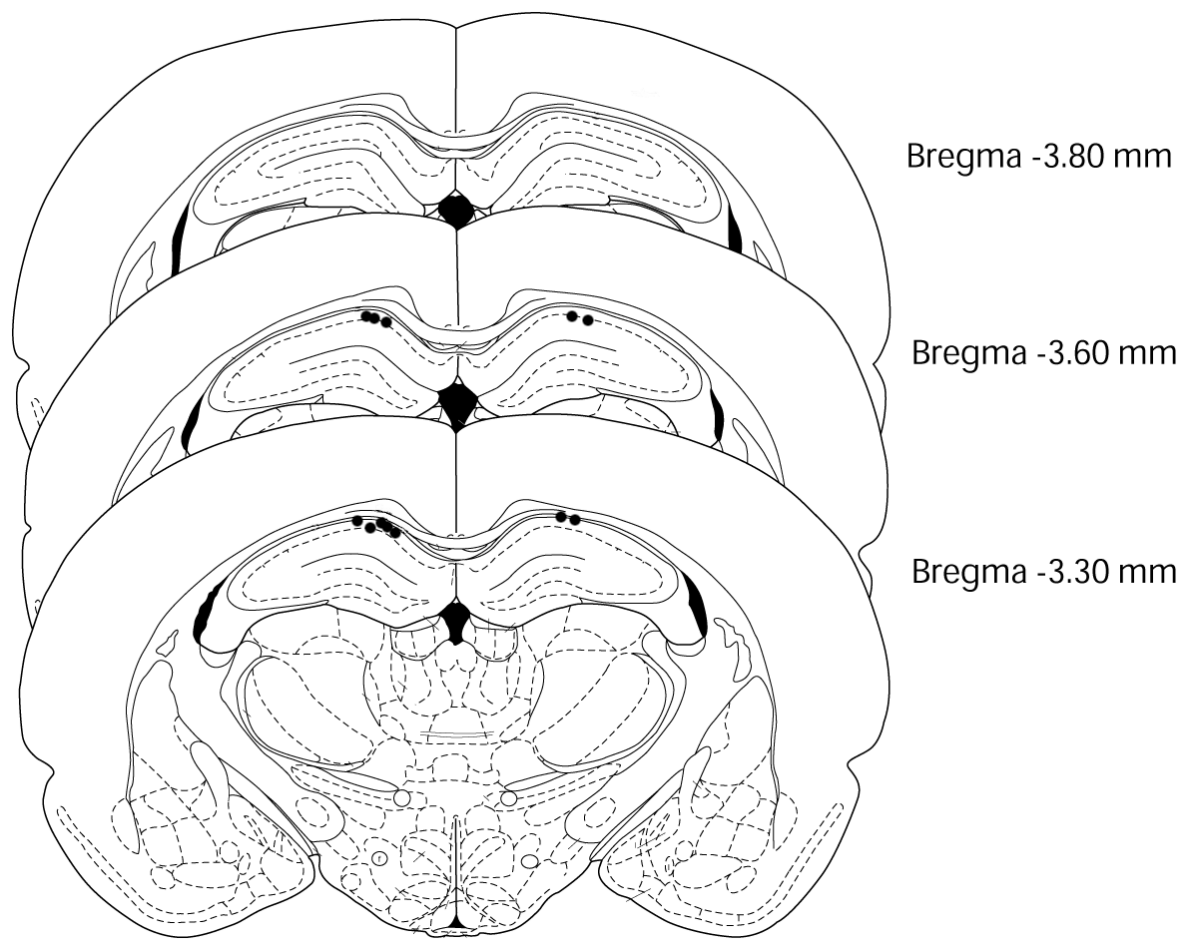
Electrode placements in the rat brains. This figure was modified from “the rat brain in stereotaxic coordinates” of Paxinos and Watson (1997). Black circles indicate the placement of the electrode tips in each rat (*n* = 12).

At the end of the experiment, all rats were deeply anesthetized with an overdose of thiamylal sodium (100 mg/kg i.p.) and perfused with saline, followed by treatment with 10% buffered formalin solution. After the brains were removed, they were post-fixed for 24 h in 10% buffered formalin, and then soaked in 30% sucrose in phosphate-buffered saline. The brains were then frozen and sectioned at 30-µm thickness. We analyzed the unilateral hippocampal EEG (Figure 2). Each rat had 2 EEG electrodes in the hippocampus: one in the right and the other in the left hemisphere. We only analyzed the data from the electrode that had the greater amplitude of theta oscillations.

## Results

### Behavior

**Figure 3:**
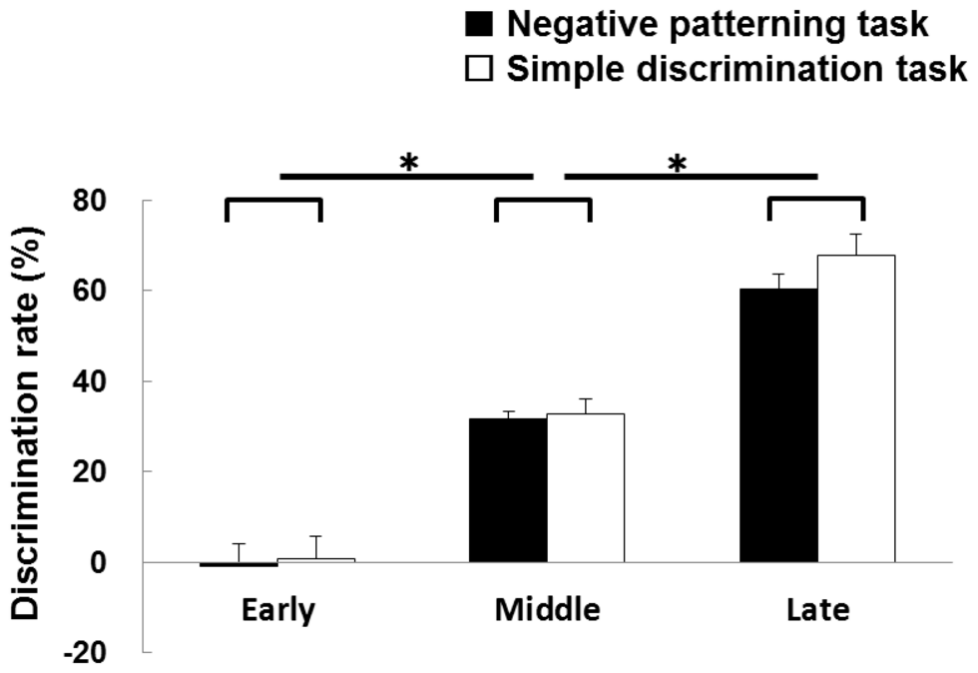
The mean discriminative rate on early, middle and late of learning stage. Statistical analysis confirmed that the discriminative rate was the highest in the late stage, followed by middle and early stages in both tasks (*: *p* < 0.05).

Learning was considered complete when the criteria were achieved for 2 consecutive days, discriminative rate was ≥ 50%. The mean number of sessions required to reach full learning criteria were 25.17 ± 2.85 (mean ± S.E.M.) and 9.50 ± 2.43 for the negative patterning and simple discrimination tasks, respectively. The difference in response rate between RFTs and non-RFTs during the early, middle, and late stages were -0.83 ± 4.92, 31.67 ± 1.61, and 60.28 ± 3.53%, respectively, in the negative patterning task group, and the corresponding values for the simple discrimination task were 0.83 ± 4.84, 32.78 ± 3.35, and 67.78 ± 4.63% (Figure 3). Two-way mixed ANOVA suggests that there is a significant effect of stages (early, middle, and late; *F*_(2,20)_ = 103.96, *p* < 0.001), but no significant group effect (negative patterning task and simple discrimination task groups; *F*_(1,10)_ = 0.19, *n.s.*) or no stage × group interaction (*F*_(2,20)_ = 0.40, *n.s.*). *Post-hoc* tests revealed that the discriminative rate was the highest in the late stage, followed by middle and early stages, in both tasks (*p* < 0.05).

The mean lever press response reaction time for stimulus in RFTs and non-RFTs at each stage (early, middle, and late) were 3.03 ± 0.18 s at the early stage, 3.15 ± 0.31 s, 1.80 ± 0.08 s at the middle stage and 3.37 ± 0.16 s, and 1.62 ± 0.14 s, and 4.11 ± 0.35 s at the late stage in the negative patterning task. The mean lever press response reaction time for stimulus in RFTs and non-RFTs at each stage (early, middle, and late) were 2.95 ± 0.70 s and 2.81 ± 0.66 s at the early stage, 3.34 ± 0.56 s and 3.77 ± 0.20 s at the middle stage, and 3.03 ± 0.35 s and 3.77 ± 0.45 s at the late stage in the simple discrimination task.

### Hippocampal theta activity

**Figure 4:**
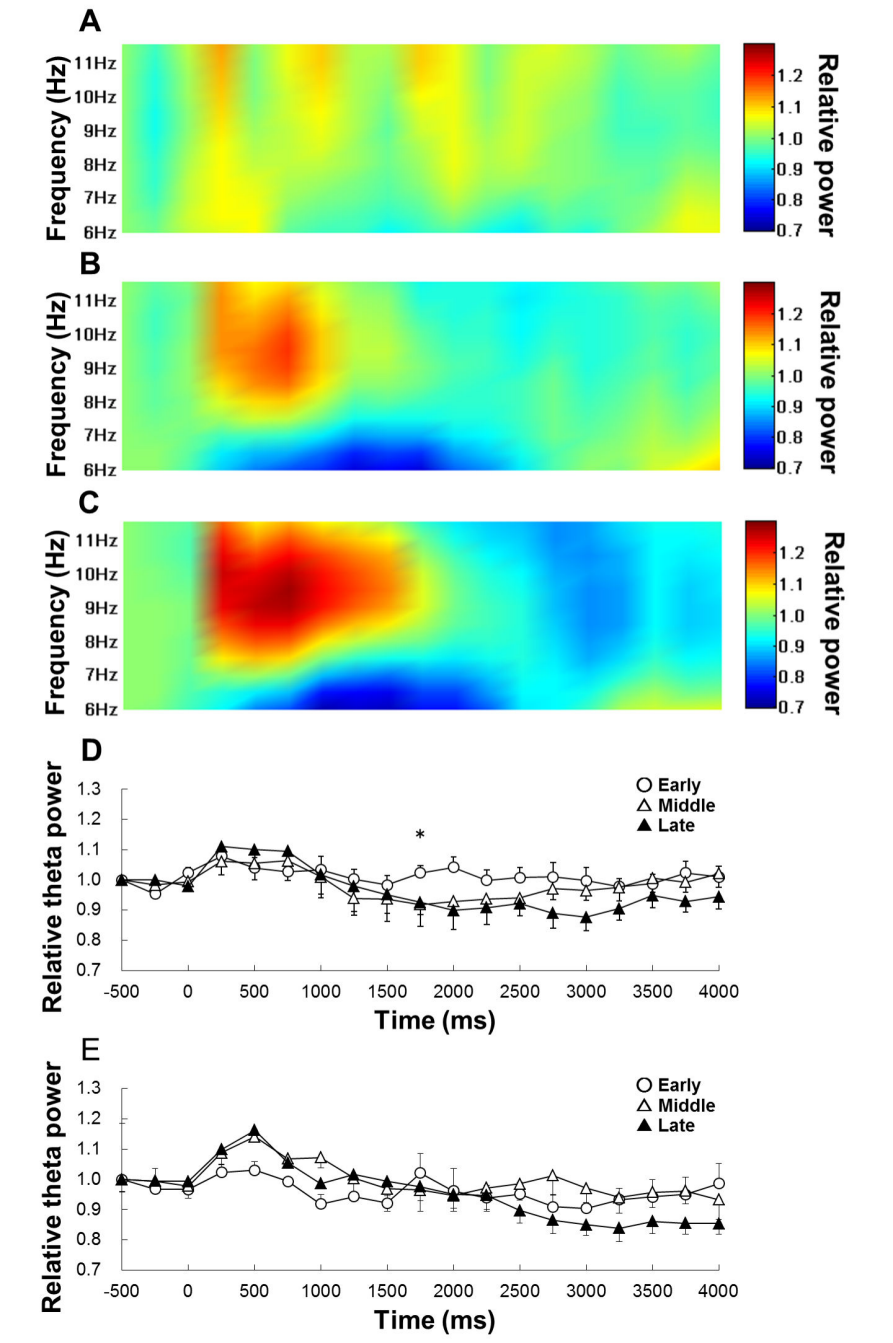
The change in theta power during the RFTs duringeach learning stage of the negative patterning task. Panel A shows the change in hippocampal theta activity along a time course during RFTs on the early stage, panel B shows theta activity on the middle stage and panel C shows theta activity on late stage of negative patterning task. The x-axis is time (ms) and the y-axis is frequency (Hz). In each panel, the period is from 500 ms before stimulus onset to 4000 ms after stimulus onset. The period was divided into 19 sub-periods of 250 ms each. The mean hippocampal theta power during 500 ms before stimulus onset was counted as the -500-ms period (no stimuli were present and no rats pressed the lever during this period) and the relative theta power calculated for each period was normalized to that during the -500-ms period (relative theta activity of each period = theta power of each period/theta power at the -500-ms period). Panel D contains a comparison of the mean (± S.E.M.) relative hippocampal theta activity at 6–12 Hz among each learning stage (early, middle, and late) throughout the time course of the experiment during RFT of the negative patterning task. Panel E contains a comparison of the mean (± S.E.M.) relative hippocampal theta activity at 6–12 Hz among each learning stage (early, middle, and late) throughout the time course of the experiment during RFT of the simple discrimination task.

First, we examined the change of relative hippocampal theta power on each learning stage of the negative patterning task. Two-way within-subjects ANOVA suggests that there is a significant interaction of learning stages (early, middle, late) × 19 epochs (-500–4000 ms, with each 250 ms; *F*_(36,180)_ = 1.68, *p* < 0.05) and effect of epochs (F_(18,90)_ = 0.3.42, *p* < 0.05; Figure 4), but no significant effect of stages (*F*_(2,10)_ = 6.00, *n.s.*) on relative hippocampal theta power during RFTs of the negative patterning task (Figure 2). *Post-hoc* tests showed that there was a significant simple main effect on the 1750-ms epochs during RFTs. Multiple comparisons revealed that hippocampal theta power increased in the 1750-ms epochs during RFTs in the early stage compared with the middle and late stage (*p* < 0.05; Figure 4). Next, the analysis was focused on the epochs (1750-ms periods) and we compared the hippocampal theta power between the negative patterning and simple discrimination tasks. Two-way mixed ANOVA suggests that there is no significant interaction of learning stages (early, middle, late) ×groups (negative patterning task and simple discrimination task groups; *F*_(2,20)_ = 1.73, *n.s.*).

**Figure 5:**
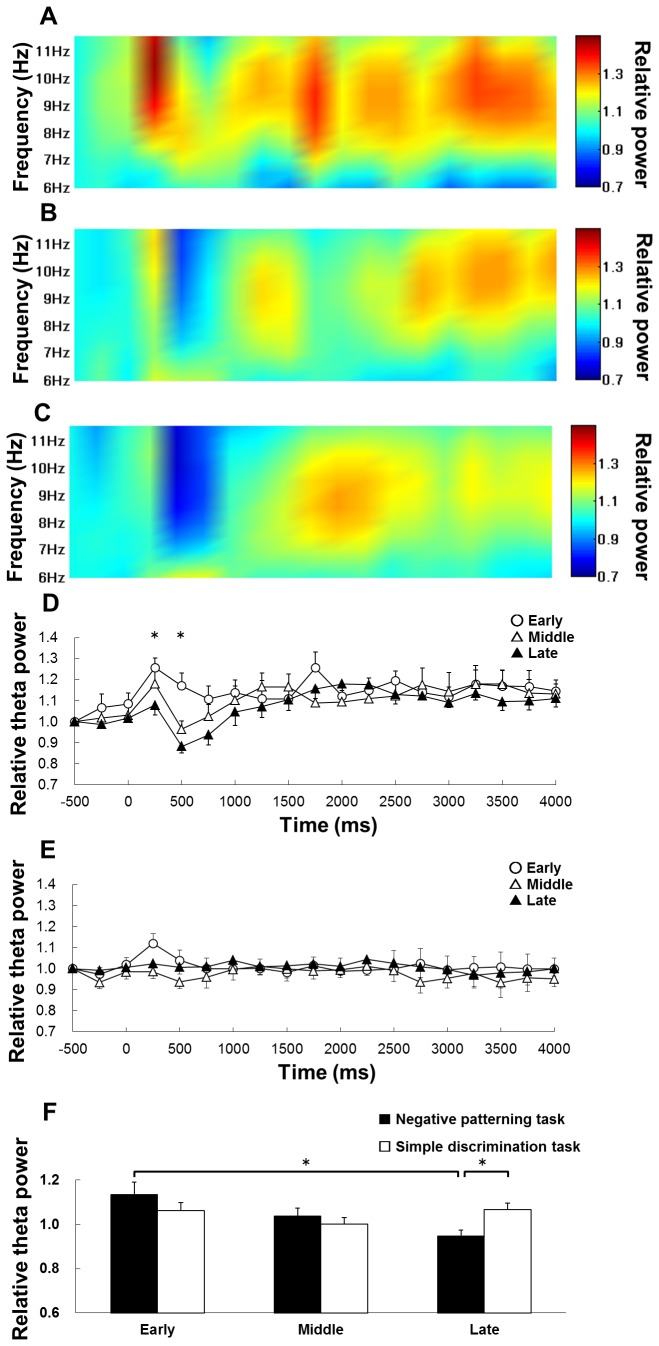
The change in theta power during the non-RFTs during each learning stage of the negative patterning task. Panel A shows the change in hippocampal theta activity along a time course during non-RFTs on the early stage, panel B shows theta activity on the middle stage and panel C shows theta activity on late stage of negative patterning task. The x-axis is time (ms) and the y-axis is frequency (Hz). In each panel, the period is from 500 ms before stimulus onset to 4000 ms after stimulus onset. The period was divided into 19 sub-periods of 250 ms each. The mean hippocampal theta power during 500 ms before stimulus onset was counted as the -500-ms period (no stimuli were present and no rats pressed the lever during this period) and the relative theta power calculated for each period was normalized to that during the -500-ms period (relative theta activity of each period = theta power of each period/theta power at the -500-ms period). Panel D contains a comparison of the mean (± S.E.M.) relative hippocampal theta activity at 6–12 Hz among each learning stage (early, middle, and late) throughout the time course of the experiment during non-RFT of the negative patterning task (*: *p* < 0.05). Panel E contains a comparison of the mean (± S.E.M.) relative hippocampal theta activity at 6–12 Hz among each learning stage (early, middle, and late) throughout the time course of the experiment during non-RFT of the simple discrimination task.

Two-way within-subjects ANOVA suggests that there is a significant interaction of learning stages (early, middle, late) × epochs (-500–4000 ms, with each 250 ms; *F*_(36,180)_ = 2.37, *p* < 0.05) and a significant effect of epochs (*F*_(18,90)_ = 4.80, *p* < 0.05), but no significant effect of stages (*F*_(2,10)_ = 0.97, *n.s.*) on relative hippocampal theta power during non-RFTs of the negative patterning task (Figure 5). *Post-hoc* tests showed that there was a significant simple main effect in the 250- and 500-ms epochs during non-RFTs. Multiple comparisons revealed that hippocampal theta power decreased in the 250-ms epochs during non-RFTs in the late stage compared with the early stage (*p* < 0.05) and in the 500-ms epochs during non-RFTs in the middle and late stages compared with the early stage (*p* < 0.05).

**Figure 6:**
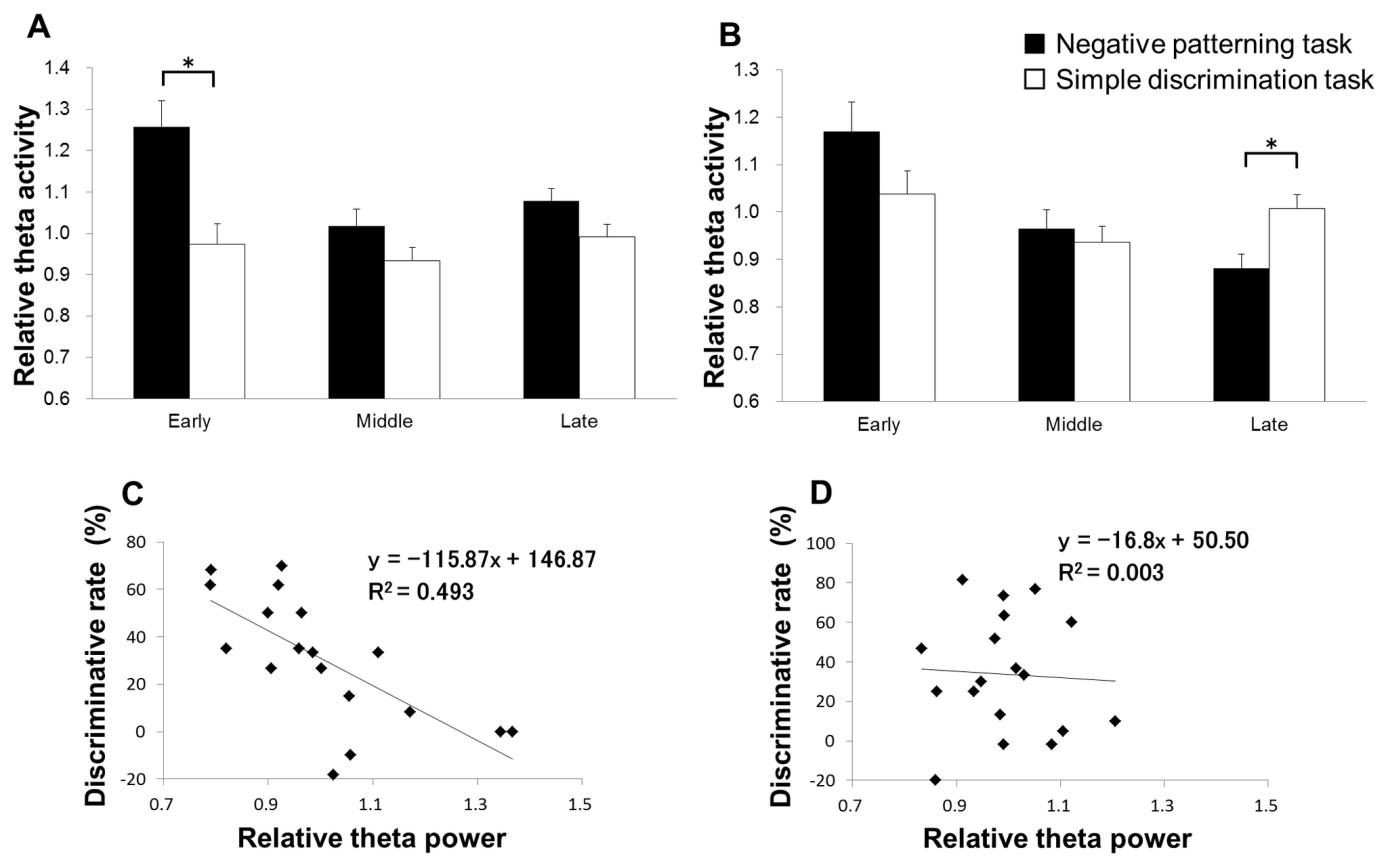
A comparison of the mean relative hippocampal theta activity between tasks. Panel A shows the relative hippocampal theta power during the 250-ms and Panel B does during 500-ms epochs between the negative patterning and simple discrimination task groups. A group (negative patterning task and simple discrimination task groups) × stage (early, middle, and late) ANOVA for hippocampal theta activity during a 250-ms epoch in the non-RFTs showed a significant interaction (*F*_(2,20)_ = 5.18, *p* < 0.05). Multiple comparisons revealed that hippocampal theta power increased during the early stage in the negative patterning task compared to the simple discrimination task group (*p* < 0.05). A group (negative patterning task and simple discrimination task groups) × stage (early, middle, and late) ANOVA for hippocampal theta activity during a 500-ms epoch in the non-RFT showed a significant interaction (*F*_(2,20)_ = 6.12, *p* < 0.05). Multiple comparisons revealed that hippocampal theta power decreased during the late stage in the negative patterning task compared to the simple discrimination task group (*p* < 0.05; *: *p* < 0.05). Hippocampal theta power during the 500 ms non-RFT correlated with the discrimination rate in the negative patterning task (*r* = -0.70, *p* < 0.05; panel C), but not the simple discrimination task (*r* = -0.06, *p* = *n.s*; panel D).

Next, we compared hippocampal theta activity between the negative patterning and simple discrimination tasks. The analysis was focused on each epoch (250- and 500-ms periods). Two-way mixed ANOVA suggests that there is a significant interaction of learning stages (early, middle, late) × groups (negative patterning task and simple discrimination task groups; *F*_(2,20)_ = 5.18, *p* < 0.05) and a significant effect of stage (*F*_(2,20)_ = 7.75, *p* < 0.05) and group (*F*_(1,10)_ = 22.28, *p* < 0.05) on hippocampal theta activity during the 250-ms non-RFT epoch. Multiple comparisons revealed that hippocampal theta power decreased during the late stage compared with the early stage (*p* < 0.05; Figure 6) in the negative patterning task. Moreover, hippocampal theta power increased in the early stage of the negative patterning task compared with that of the simple discrimination task (*p* < 0.05; Figure 6). However, there was no significant difference in hippocampal theta power during the late stage between the negative patterning and the simple discrimination tasks. Two-way mixed ANOVA suggests that there was a significant interaction of learning stages (early, middle, late) × groups (negative patterning task and simple discrimination task groups; *F*_(2,20)_ = 6.12, *p* < 0.05) and a significant effect of stage (*F*_(2,20)_ = 12.00, *p* < 0.05) and group (*F*_(1,10)_ = 0.07, *n.s.*) on hippocampal theta activity during the 500-ms non-RFT epoch. Multiple comparisons revealed that hippocampal theta power decreased during the late stage compared with the early stage (*p* < 0.05; Figure 6) in the negative patterning task. Furthermore, hippocampal theta power decreased during the late stage of the negative patterning task compared with that of the simple discrimination task (*p* < 0.05; Figure 6). Hippocampal theta power during the 500-ms non-RFT epoch correlated with the discrimination rate in the negative patterning task (*r* = -0.70, *p* < 0.05; Figure 6), but not the simple discrimination task (*r* = -0.06, *p* = n.s.; Figure 6).

**Figure 7:**
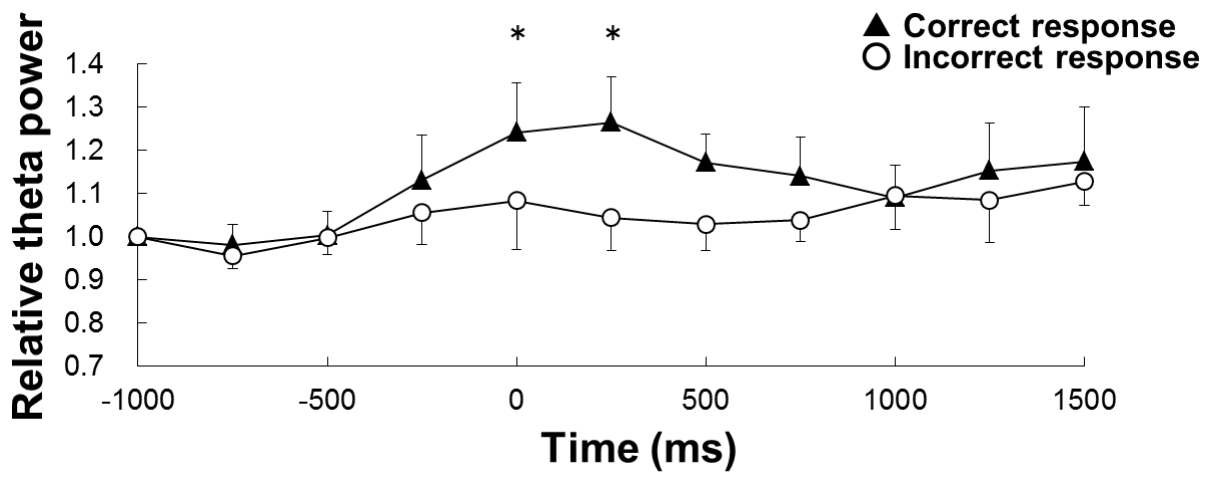
Comparison of hippocampal theta power between correct-response and incorrect-response trials. This figure shows the hippocampal theta power between trials with correct lever press response for RFT and incorrect lever press responses for non-RFTs during the late stage of the negative patterning task. The 0 period was lever press timing. The analysis period from 1250 ms before lever press to 1500 ms after lever press was divided into 11 250-ms epochs. The 250-ms period from -1250 to -1000 ms was used as the baseline, and the relative theta activity for each period was calculated as follows: relative theta activity of each period = theta activity of each period/theta activity during the baseline period.

Finally, we compared the hippocampal theta power between trials with correct lever press response for RFT and incorrect lever press responses for non-RFTs during the late stage of the negative patterning task (Figure 7). The analysis period from 1250 ms before lever press to 1500 ms after lever press was divided into 11 250-ms epochs. We considered the 250-ms pre-lever press timing period (from -1250 to -1000 ms) as baseline, and the relative theta activity was calculated for each period as follows: relative theta activity of each period = theta activity during each period/theta activity during baseline. Two-way within-subjects ANOVA suggests that there is a significant interaction of trial type (correct lever press response for RFT and incorrect lever press response for non-RFT) × periods (-1000 ms -1500 ms; *F*_(10,50)_ = 2.12, *p* < 9.05) and a significant effect of epochs (*F*_(10,50)_ = 2.15, *p* < 0.05), but no significant effect of trial type (*F*_(1,5)_ = 4.18, *n.s.*). *Post-hoc* tests showed that there was a significant difference during the 0 to 250-ms epoch.

## Discussion

### Hippocampal theta activity during acquisition of the stimulus discrimination task

This study examined the change in hippocampal theta activity during the acquisition of a negative patterning task and compared the hippocampal theta power between the negative patterning and a simple discrimination task. The results showed that hippocampal theta activity during the early stage of learning of the negative patterning task increased transiently during non-RFTs compared with the simple discrimination task. We think that this difference in theta activity probably reflects the difference in attention being paid to the different stimuli presented during the 2 tasks. In the negative patterning task, a cross-modal compound stimulus was presented during non-RFTs, but in the simple discrimination task, a single modality stimulus was presented during non-RFTs. Moreover, the difference in hippocampal theta activity was only observed in the early stage of learning but not in the late stage. This result suggests the possibility that the rats became habituated to the presented stimulus during task acquisition. Thus, the increase in theta activity during the 250-ms non-RFT epoch of the negative patterning task might reflect a difference in attention to the cross-modal feature or intensity of the stimulus.

Hippocampal theta power declined transiently in the 500-ms non-RFT epoch during the late stage of learning for the negative patterning task compared with the simple discrimination task. Several studies have examined changes in hippocampal theta activity during acquisition of learning tasks [6,8,9,15,16]. Grastyán et al. [6] examined the relationship between hippocampal theta activity and acquisition of an orientative conditioned response (CR) for a tone stimulus presentation. Their results showed that although the cats’ hippocampal theta activity increased with the acquisition of the association between stimulus and orientative CR, the hippocampal theta wave decreased after formation of this association. Thus, it is conceivable that the transient decrease of hippocampal theta activity during the late stage of learning in the negative patterning task observed in the current study may be related to the mastery of the negative patterning task. However, this interpretation does not explain the greater decrement of hippocampal theta activity in the late stage of learning of the negative patterning task than of the simple discrimination task. We believe that this difference in the decrement of hippocampal theta activity between these tasks may be attributed to differences in specific features of these tasks.

### Inhibitory learning and the negative patterning task

In this study, the difference in the hippocampal theta activity was observed during non-RFTs but not during RFTs. In non-RFTs, rats needed to learn a no-go response at the presentation of previously reinforced stimuli. Recently, several studies have proposed that hippocampal function is important in the acquisition of inhibitory learning [1,2,17]. According to the hypothesis of inhibitory learning, a go response for a stimulus is elicited by an excitatory association between the stimulus and reward. In order to elicit a no-go response for the same stimulus, a more intense inhibitory association between the stimulus and reward is required. Thus, it has been proposed that the hippocampus is needed to form simple inhibitory associations between events concurrently embedded as simple excitatory associations [1,2,17]. Chan et al. [1] postulated this idea from evidence of extinction learning in rats. In their study, an animal was initially trained to respond for a stimulus (S+); subsequently, in the extinction phase, the animal was trained to withhold responses for the same stimulus (S-). These authors discussed that extinction of a stimulus-reward association that was given prior excitatory training results in the formation of an inhibitory association, and that the excitatory and inhibitory associations then exist concurrently; thus, response inhibition requires an inhibitory association that is more intense than the previously paired excitatory association [1]. In the same study, ibotenate-induced hippocampal lesions impaired extinction learning, suggesting that the hippocampus is important for the formation of inhibitory associations between a stimulus and reward. In addition, the authors proposed that a negative patterning task has features similar to the extinction learning task. The compound stimulus of a negative patterning task consists of single stimuli that are associated with reinforcement; thus, the compound stimulus of a negative patterning task has a latent excitatory association with reward. Therefore, to correctly respond to the compound stimulus of a negative patterning task, the rat is required to form a more intense inhibitory association to override this excitatory association.

On the other hand, several studies have shown that hippocampal theta activity is affected by motor activity in rats [18–22]. Vanderwolf [21] demonstrated that hippocampal theta activity is related to voluntary movement of different types. In rats, immobility-associated theta activity is less obvious, but is seen when the rat is sniffing [18] or preparing to jump [22]. Additionally, some studies of hippocampal theta waves have suggested the possibility that a decline of hippocampal theta activity may occur during behavioral inhibition [19,20]. Sinnamon [19] showed that hippocampal theta activity decreased when rats stopped in the middle of an approach to a reward. This author proposed that this cessation or inhibition of a go response induced the observed decline of hippocampal theta activity. In accordance with this, we believe that the transient decline of the hippocampal theta activity that was observed in the current study may reflect inhibitory learning or response inhibition.

### Relationship between hippocampal theta activity and movement during RFTs and non-RFTs

Several researchers have shown that hippocampal theta activity is strongly related to voluntary motor movements in rats, such as running, jumping, rearing, exploratory behavior, sniffing, and lever pressing [21,22]. In contrast, another study showed that hippocampal theta rhythm was related to the approach behavior induced by reward memory [23]. The results of this study indicated that hippocampal theta power would increase during the 500-ms periods during the middle and late stages of learning, but a significant difference from baseline measures. In the current study, rats performed a lever press movement for reinforced stimulus in RFTs in order to gain the reward. The reaction time of the lever press movement for the stimulus reward in RFTs was about 2 s. On the other hand, rats did not perform lever press movements during non-RFTs. As far as we observed, there was no difference in the rats’ behavior during the late stage of learning between the task groups. During the non-RFTs, most rats were resting or waiting in front of the lever. Thus, we believe that the difference in hippocampal theta activity during the late stages of learning between the negative patterning and simple discrimination tasks was not caused by any differences in movement.

Recently, Wyble et al. [24] revealed that there was a difference in hippocampal theta activity between behaviors that were associated with reward and those that were not associated with reward. They reported that hippocampal theta power showed a greater transient decline during lever presses that were not associated with a reward than those that were associated with a reward. The investigators claimed that this transient decline in hippocampal theta power related to the expectation of no reward. In the present study, the decrease in hippocampal theta power was greater during incorrect lever press responses for non-RFT than during correct lever press responses for RFT in the late stage of the negative patterning task. A lever press response for RFT was associated with a reward but a lever press response for non-RFT was not. These results are consistent with the previous study [24], which found that the transient decline in hippocampal theta power was greater during lever presses that did not yield a reward than those that did yield a reward. Thus, the transient decline in hippocampal theta power that was observed in this study might relate to the rats’ expectation of reward or no reward.

### Neural mechanism of the decline of the hippocampal theta activity

Hippocampal theta power is affected by activity of cholinergic and gamma aminobutyric acid (GABA) ergic neurons of the medial septal/diagonal band area [25]. Monmaur and Breton [26] showed that theta activity increased when the cholinergic agonist, carbachol, was injected into the intraseptum in freely moving rats. Thus, it may be possible that the transient decrease of hippocampal theta observed in the current study is due to a transient decrease in the septal cholinergic activity. On the other hand, Allen and Crawford [27] reported that hippocampal theta activity decreased when the GABA agonist muscimol was injected into the medial septum in rats. Hence, we propose that the transient decrease of hippocampal theta activity during compound stimulus learning in the negative patterning task is induced by the activity of septal cholinergic or GABAergic neurons, or their interaction. In future studies, the relationship between the negative patterning task and septal cholinergic and/or GABAergic activity should be examined.

## Conclusion

The hippocampus plays an important role in the no-go response to a conflicting stimulus with incompatible goals or response tendencies. This study examined hippocampal theta activity during a no-go response to a stimulus with conflict by using a negative patterning task. The results showed a transient decline of hippocampal theta power during a 500-ms epoch of acquisition of a negative patterning task. This transient decline in hippocampal theta power was greater in the late stage of the negative patterning task than in the late stage of the simple discrimination task. Recently, Sakimoto et al. [28] demonstrated a transient decline in hippocampal theta power during a no-go response in the reversal phase of non-RFTs compared with that in the discrimination phase of a discrimination-reversal task. In this task, animals first learn to emit the go response to one stimulus and the no-go response to another stimulus (S1+, S2−) during the discrimination phase, and then they learn to reverse these relationships between stimulus and response during the reversal phase (S1-, S2+). S1 was previously associated with a go response and, therefore, the stimulus had simple inhibitory associations between events concurrently embedded in simple excitatory associations during the reversal phase. Thus, we propose that the transient decline in hippocampal theta power observed in this study might relate to the formation of an inhibitory response to a conflicting stimulus with incompatible goals or response tendencies.
